# Diversifying the Flavor of Black Rice Wines through Three Different Regional Xiaoqus in China and Unraveling Their Core Functional Microorganisms

**DOI:** 10.3390/foods12193576

**Published:** 2023-09-26

**Authors:** Aoxing Tang, Bangzhu Peng

**Affiliations:** 1 College of Food Science and Technology, Huazhong Agricultural University, Wuhan 430070, China; tangaoxinghn@163.com; 2 Hubei Key Laboratory of Fruit & Vegetable Processing & Quality Control, Huazhong Agricultural University, Wuhan 430070, China; 3 Shenzhen Institute of Nutrition and Health, Huazhong Agricultural University, Wuhan 430070, China; 4 Shenzhen Branch, Guangdong Laboratory for Lingnan Modern Agriculture, Genome Analysis Laboratory of the Ministry of Agriculture, Agricultural Genomics Institute at Shenzhen, Chinese Academy of Agricultural Sciences, Shenzhen 518120, China

**Keywords:** black rice wine, Xiaoqu, characteristic flavor compounds, core microbes, correlation analysis

## Abstract

The flavor of black rice wine (BRW) can be diversified by the Xiaoqus, from different regions; however, the functional microbiota that contributes to its flavor remains unclear. Accordingly, this study selected three regional Xiaoqus from Sichuan Dazhu (Q1), Jiangxi Yingtan (Q2), and Hubei Fangxian (Q3) as starters to investigate flavor compounds and microbial communities during BRW brewing. Results indicated that altogether 61 flavor substances were identified, 16 of which were common characteristic flavor compounds (odor activity value > 0.1). Each BRW possessed unique characteristic flavor compounds. O2PLS and Spearman’s correlation analysis determined that characteristic flavor compounds of BRW were mainly produced by *Saccharomyces cerevisiae*, non-Saccharomyces yeasts, and lactic acid bacteria, with the common core functional strains being Wickerhamomyces and Pediococcus, and with their unique core functional strain likely causing a unique characteristic flavor. This study could promote the high-quality development of the black rice wine industry.

## 1. Introduction

Black rice wine (BRW), a traditional Chinese yellow rice wine, is brewed from black rice with abundant anthocyanins. It has an attractive appearance, unique aroma, and numerous bioactive components [1,2]. The black rice bran, containing abundant anthocyanins, has a hard shell composed of pectin and cellulose [3], making it difficult to cook and leading to an extensive loss of anthocyanins during the soaking and cooking processes of traditional BRW brewing. During the fermentation process, the black rice bran acts as a barrier, reducing the decomposition efficiency of amylase and protease on the substrate, which in turn decreases the production of flavor compounds and adversely affects the quality of BRW [4]. With the heightened attention to black rice, black rice wine, and other related products in recent years [3,5], the obstacles in the traditional BRW brewing process have been remarkably improved. Black rice is properly milled to obtain polished black rice and black rice bran, and then the subsequent brewing process [6] can significantly improve the quality of BWR such as giving a higher anthocyanin content and better flavor.

Apart from its appealing color and nutritious content, the distinctive flavor of BRW is what makes it so appealing to consumers [7]. Commonly, esters, alcohols, aldehydes, ketones, acids and phenols make up the volatile profiles of BRW. Moreover, Yu et al. concluded that the characteristic aromatic substances of Chinese traditional rice wine were ethyl butyrate, nonanal, ethyl octanoate and ethyl phenylacetic acid, with low content and high odor activity value (OAV) [8]. It has been reported that the primary flavor components of BRW are alcohols and esters [9], yet its flavor is distinct from that of traditional glutinous rice wine. This is due to the difference in raw materials, leading to differences in the characteristic aroma components of rice wine [7]. Nevertheless, the characteristic flavor compounds of BRW have yet to be determined.

Microbial metabolism is indispensable for the aroma formation of fermented foods. Identifying microorganisms responsible for key volatile compound formation would be beneficial in understanding and improving the fermentation process [10]. Zhao et al. utilized two-way orthogonal partial least squares (O2PLS) analysis to identify 12 microbial genera, such as *Rhizopus*, *Aspergillus* and *Pediococcus*, which were strongly associated with the majority of aromatic substances in Guizhou traditional black glutinous rice wine [9]. Additionally, they found that inoculation of Rhizopus and Saccharomyces cerevisiae could significantly enhance the final product aroma [11]. Jiang et al. selected two traditional black glutinous rice wine starters from Guizhou as research objects and demonstrated that lactic acid bacteria (LAB) could be a major factor in flavor production, using Spearman’s correlation [12]. Cao et al. found a significant variation in the predominant microbes due to the geographical difference, thus resulting in different flavors and tastes of rice wine [13]. Research on BRW is currently restricted to the Guizhou region, thus resulting in a single category. Hence, to enrich the diversity of BRW’s flavor, it is necessary to brew different regional Xiaoqus of BRW and further investigate the characteristic aromatic substances and core functional microbial communities. 

In this study, microbial community structures and volatile flavor compounds were dynamically monitored by high-throughput sequencing (HTS) and headspace solid-phase microextraction (HS-SPME), coupled with gas chromatography–mass spectrometry (GC/MS) analysis. The similarities and distinctions in characteristic flavor compounds were identified among three BRW, via OAV. O2PLS analysis enabled the identification of the microorganisms associated with the majority of volatile components, while Spearman’s correlation was used to determine the strains responsible for producing the characteristic aroma compounds, with common strains being the core functional microbial communities. This research result could not only reveal the characteristic flavor compounds of BRW, but also help us to develop special starters for BRW, with varied flavors. 

## 2. Materials and Methods

### 2.1. Collection of Xiaoqu Samples and Black Rice 

Three representative samples of Xiaoqu starters were selected from Dazhu, Sichuan Province (Q1), Yingtan, Jiangxi Province (Q2), and Fang County, Hubei Province (Q3), China. Black rice, the Huamoxiang No. 5 variety provided by the College of Plant Science, Huazhong Agricultural University, was properly milled to obtain polished black rice and black rice bran (the proportion being 13%), using a CN-300R Rice Milling Machine (Korea New Agricultural Industrial Co. Ltd., Gimcheon, Korea). The Xiaoqu samples and raw materials were chilled at 4 °C during transport and storage.

### 2.2. BRW Fermentation and Sample Collection

In the manufacturing processes of BRW, 300 g of polished black rice were washed and soaked in water overnight to ensure sufficient water absorption. After the polished black rice was drained, it was steamed under normal pressure for 1 h, and then cooled to room temperature. Following this, the mixture was mixed with cooled rice, 45 g of steam-sterilized black rice bran, 12 g of Xiaoqu powder, and sterile water (material–liquid ratio of 1:1.1), and was transferred to a 1 L triangular flask, sealed at the mouth with a vented sealing film, and underwent saccharification at 30 °C for 3 days (by conducting preliminary experiments, we could determine the maximum reducing-sugar content on the third day). After that, sterile water was added to attain a 1:1.8 ratio of the material liquid. Then the triangular flasks were sealed with a one-way valve and the mixture continued to undergo alcoholic fermentation at 28 °C for 4 days, after which it was transferred to 20 °C for a further 7 days. Triplicate independent brewing was conducted for each Xiaoqu.

At 0, 36, 72 168 and 336 h, 20 g of the fermentation mixture was randomly collected under aseptic conditions and centrifuged. The supernatant was used for determining physicochemical properties and flavor profiles, and the sediment was stored at −80 °C for microbiota analysis using high-throughput sequencing (HTS).

### 2.3. Physiochemical Property Determination

Physiochemical properties, including reducing sugar, alcohol, total acidity and pH were tested according to the Chinese national standard of GB/T 13662-2018. The anthocyanin content was analyzed according to the method of Wrolstad et al. [14]. All sample analyses were carried out in triplicate.

### 2.4. Measurement of Flavor Compounds 

Flavor compounds in all samples were measured using headspace solid-phase microextraction coupled with gas chromatography–mass spectrometry (HS-SPME-GC-MS). Briefly, each sample (5 mL) was enclosed in a 20 mL SPME glass vial with 1.2 g of NaCl and 3 μL of the internal standard 2-octanol (328.8 μg/mL in absolute chromatographic grade ethanol). A DVB/CAR/PDMS fiber (Supelco, Bellefonte, PA, USA) was used to extract flavor compounds at 45 ℃ for 45 min. The compounds were desorbed and detected via GC-MS (7000D; Agilent Technologies, Santa Clara, CA, USA) equipped with an HP-5MS column (30 m × 250 μm × 0.25 μm; Agilent Technologies, USA). The initial temperature of the oven was held at 40 °C for 2 min, and then increased to 140 °C at 2 °C/min, heated at 10 °C/min to 230 °C, and held for 5 min. The ion energy for the electron impact (EI) was kept at 70 eV and used a full scan ranging from 40 to 500 amu. The constituents were identified by matching the mass spectrum with the NIST14 and Kovats retention indices (RI). Semi-quantitative compound analysis was performed by comparing the area with the internal standard [12]. The odor activity value (OAV) was calculated according to OAV = C/T (C was the concentration of flavor compounds; T was the odor threshold of the compound). All sample analyses were carried out in triplicate.

### 2.5. DNA Extraction and PCR Amplification

Total microbial genomic DNA was extracted from the BRW samples using the E.Z.N.A.^®^ soil DNA Kit (Omega Bio-tek, Norcross, GA, USA) according to the manufacturer’s instructions. The hypervariable region V3-V4 of the bacterial 16S rRNA gene was amplified with primer pairs 338F (5′-ACTCCTACGGGAGGCAGCAG-3′) and reverse primer 806R (5′-GGACTACHVGGGTWTCTAAT-3′), and the ITS1 region of the fungi was amplified with the forward primer ITS1F (5′-CTTGGTCATTTAGAGGAAGTAA-3′) and reverse primer (5′-GCTGCGTTCTTCATCGATGC-3′) by an ABI GeneAmp^®^ 9700 PCR thermocycler (ABI, Foster City, CA, USA). The polymerase chain reaction (PCR) amplification cycling conditions were established by referring to Yan et al. [13]. The PCR product was extracted from 2% agarose gel, purified using the AxyPrep DNA Gel Extraction Kit (Axygen Biosciences, Union City, CA, USA), and quantified using Quantus™ Fluorometer (Promega, Madison, WI, USA).

### 2.6. Illumina MiSeq Sequencing and Bioinformatic Analysis 

Purified amplicons were pooled in equimolar amounts and paired-end sequenced on an Illumina MiSeq PE300 platform (Illumina, San Diego, CA, USA), according to the standard protocols, by Majorbio Bio-Pharm Technology Co. Ltd. (Shanghai, China). The raw sequencing reads were deposited in the NCBI Sequence Read Archive (SRA) database (Accession Number: PRJNA984427).

Raw FASTQ files were de-multiplexed, quality-filtered by FASTP version 0.19.6, and merged using FLASH version 1.2.7. Then the optimized sequences were clustered into operational taxonomic units (OTUs) using UPARSE 7.1 with a 97% sequence similarity level. The OTU table was manually filtered, i.e., chloroplast and mitochondria sequences in all samples were removed. To minimize the effects of sequencing depth on the alpha and beta diversity measure, the number of 16S and ITS gene sequences from each sample was rarefied to 22,060 and 33,759, which still yielded an average Good’s coverage of 99.09%, respectively. Alpha and beta diversity indices were analyzed using QIIME and the principal coordinate analysis (PCoA). Bioinformatic analyses (like linear discriminant analysis (LEfSe), redundancy analysis (RDA), Spearman’s correlation analysis, and so on) of the BRW microbiota were carried out using the Majorbio Cloud platform (https://cloud.majorbio.com (accessed on 11 September 2022 to 30 May 2023)). 

### 2.7. Statistical Analysis

The significance analysis was performed using the SPSS 21.0 software (IBM SPSS Inc., Chicago, IL, USA). Multivariate statistical analysis of the principal coordinate analysis (PCoA) and principal component analysis (PCA) can be carried out using SIMCA software (14.1) (UMETRICS, Malmo, Sweden). To uncover the correlation between microbiota and volatile compounds, O2PLS modeling was conducted with SIMCA software, where the microbiota data (X matrix) was linked to the volatile flavor data (Y matrix). Spearman’s correlation was then applied to explore the relationship between core microorganisms and volatile flavor compounds. Furthermore, all relevant systems were visualized through Cytoscape software (version 3.5.1) (http://www.cytoscape.com (accessed on 11 September 2022 to 30 May 2023)).

## 3. Results and Discussion 

### 3.1. Physiochemical Property Analysis 

The fermentation performance of the three BRWs exhibited marked distinctions, yet all followed similar trends during fermentation (Table S1). During fermentation, all the BRW’s total acids increased initially and then stayed constant. However, there were disparities during the early stage of alcohol fermentation (72–168 h). Specifically, Q1 increased significantly from 7.25 to 10.39 g/L, while Q2 and Q3 decreased noticeably from 7.62 and 6.91 g/L to 6.92 and 6.02 g/L, respectively. During the initial fermentation, the reducing-sugar concentration reached the highest level (Q1: 361.02, Q2: 437.16, and Q3: 325.56 g/L), due to the quick saccharification of the substrate by saccharifying enzyme-producing microorganisms [7]. Meanwhile, all groups reached their maximum alcohol content at 168 h, with no significant variance among them at the fermentation endpoint. Anthocyanins are the main active and pigment component of BRW [1], responsible for its high nutritional value and distinct appearance. During the fermentation process, the total anthocyanin content of BRW experienced a significant decrease before stabilizing. The final concentrations of Q1, Q2, and Q3 were observed to be significantly different, with values of 135.01, 77.48, and 108.29 mg/L, respectively, which may be attributed to the varying microbial composition of different Xiaoqus, resulting in dissimilar dissolution and loss of anthocyanins. The pH values were kept in a relatively low range of 4–4.6, which is beneficial for averting external contamination by miscellaneous bacteria.

### 3.2. Volatile Compound Analysis of Black Rice Wine

#### 3.2.1. Dynamic Changes in Volatile Components during Fermentation

**Figure 1:**
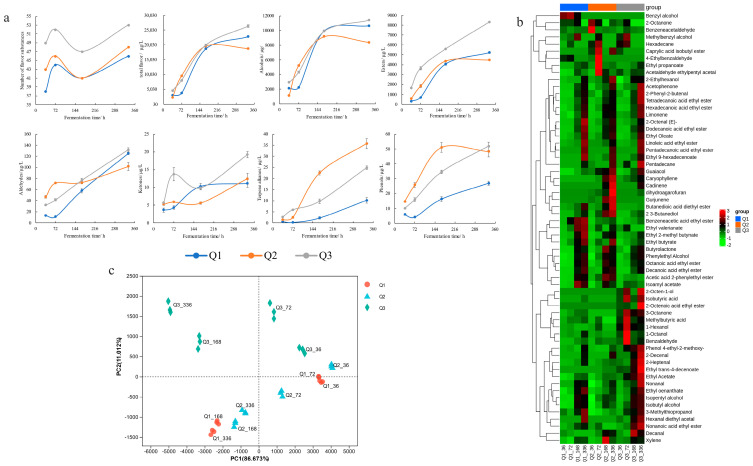
Change in volatile compounds during black rice wine fermentation. (**a**) Changes in the number of flavor substances, total flavor, alcohols, esters, aldehydes, ketones, terpene alkanes, and phenols during the black rice wine fermentation; (**b**) Heatmap visualization of the 61 volatile compounds during the black rice wine fermentation; (**c**) PCA analysis of volatile compounds among three Xiaoqu black rice wines.

A survey of BRWs uncovered 61 volatile compounds, comprising alcohols, esters, aldehydes, ketones, and phenols (Figure 1a,b) [12,15]. Over the course of fermentation, the quantity of volatile compounds in various BRWs follows a similar pattern—rising, then declining, and then rising again. The total flavor-substance content experienced a substantial rise during the early stages of alcohol fermentation (72–168 h), primarily in the form of alcohols and esters, with an increment of 3948–7667 μg/L and 1957–3387 μg/L, respectively. At the end of fermentation, 46, 48 and 53 flavor substances were identified in Q1, Q2 and Q3, respectively, with a total aroma content of 22,855.63, 18,738.32 and 26,406.41 μg/L. It has been found that the Xiaoqu of different regions has a considerable influence on wine’s volatile matter content (*p* < 0.05), which is consistent with Chen et al. [15].

Consequently, PCA analysis was utilized to further investigate the distinction in volatile compounds among the three wine samples, which showed that the first and second principal components accounted for 86.67% and 11.01%, respectively (Figure 1c). The saccharification and alcohol fermentation stages were distinguished through PC1. Moreover, Q1 and Q2 BRWs were relatively close on the plot, suggesting that their overall flavors were similar. In contrast, Q3 was situated far from Q1 and Q2 on PC2, likely due to the dissimilar microbial composition of starters in different regions [16].

#### 3.2.2. Characteristic Flavor Substances of Black Rice Wine

To further differentiate aromatic substances between different black rice wines, samples from the fermentation endpoint (336 h) were selected for orthogonal partial least squares discrimination analysis (OPLS-DA) and discriminant VIP prediction analysis (VIP_pred_ > 1 and *p* < 0.05). As depicted in Figure S1, the differential volatile components between Q1 BRW and Q2 BRW comprised isopentyl alcohol (al2), 2,3-Butanediol (al3), phenethyl alcohol (al11), ethyl acetate (et1), isoamyl acetate (et5), hexadecanoic acid ethyl ester (et22); between Q2 and Q3 they included ethyl acetate (et1), isopentyl alcohol (al2), phenethyl alcohol (al11), hexadecanoic acid ethyl ester (et22), 2,3-Butanediol (al3), isoamyl acetate (et5), butanedioic acid diethyl ester (et9); and between Q1 and Q3 they consisted of ethyl acetate (et1), isopentyl alcohol (al2), isoamyl acetate (et5), and phenethyl alcohol (al11). Results revealed that alcohols and esters, as primary differential volatile components, may contribute to the diverse aromas among different BRWs. Isoamyl alcohol, phenethyl alcohol, ethyl acetate, and isoamyl acetate were identified as common differential volatiles with higher content in BRW, and could potentially be key aromatic substances contributing to distinctive flavors. This conclusion has been echoed in other rice wines [17,18].

However, an abundance of volatile substances does not always mean that they will contribute significantly to the characteristic aroma [19]. Hence, in order to excavate the characteristic aromatic substances in BRW, an OAV was calculated for each aroma substance at the fermentation endpoint through concentration and threshold [19,20]. It is commonly accepted that compounds with OAV ≥ 1 have a major influence on the overall aroma profile, and are referred to as key characteristic flavor substances. Compounds with 1 > OAV > 0.1 have a minor effect on the overall aroma, and are known as auxiliary flavor substances. Conversely, compounds with OAV < 0.1 are generally disregarded in terms of their impact on the overall aroma. According to Table 1, there were 32 potential aroma components identified that could contribute to the characteristic flavor of three different types of BRW. The key characteristic flavor compounds (OAV ≥ 1: isoamyl acetate (et5), ethyl octanoate (et10), 2-octenal (ad5), ethyl decanoate (et17), 2-decenal (ad9), ethyl heptanoate (et8), ethyl butyrate (et3), nonanal (ad6), and guaiacol (bp2)) and auxiliary presentation flavor compounds (1 > OAV > 0.1: ethyl palmitate (et22), phenyl ethyl acetate (et13), phenyl ethanol (al11), limonene (hf1), ethyl acetate (et1), 4-ethyl guaiacol (bp3), isoamyl alcohol (al2), butyrolactone (et7), and ethyl cinnamate (et18)) have been identified. It is implied that these 18 substances may be the essential components of BRW’s characteristic aroma, thereby leading to a discrepancy in overall flavor with other rice wines. Furthermore, the key aroma compounds of these wines were mainly ethyl esters with fruit and floral aromas, which were the metabolic products of microorganisms during fermentation, rather than the raw materials themselves. Hence, to identify the core microbial community that influences the 18 characteristic flavor substances of BRW, it was essential to perform a correlation analysis between the characteristic flavor substances and the microorganisms. 

### 3.3. Microbial Analysis 

#### 3.3.1. Alpha Diversity Analysis 

The Good’s coverage values of all samples were greater than 0.999 (Table S2), demonstrating that the sequencing depth was satisfactory and could detect the vast majority of bacteria and fungi. Results from the Shannon and Chao index demonstrated that the diversity and richness of bacteria and fungi in Xiaoqu (0 h) was greater than that in the fermented samples. Additionally, the Shannon index decreased as fermentation progressed, signifying that certain microorganisms in the fermentation agent microbial community that were not conducive to the brewing environment were gradually eliminated. Overall, the ranking of microbial diversity and richness was Q2 > Q3 > Q1. During the fermentation process, both Q1 and Q3 exhibited a decrease in bacterial diversity and an increase in fungal diversity, with the latter being more abundant than the former. In contrast, Q2 revealed a higher bacterial than fungal diversity. 

#### 3.3.2. Microbial Composition

**Figure 2:**
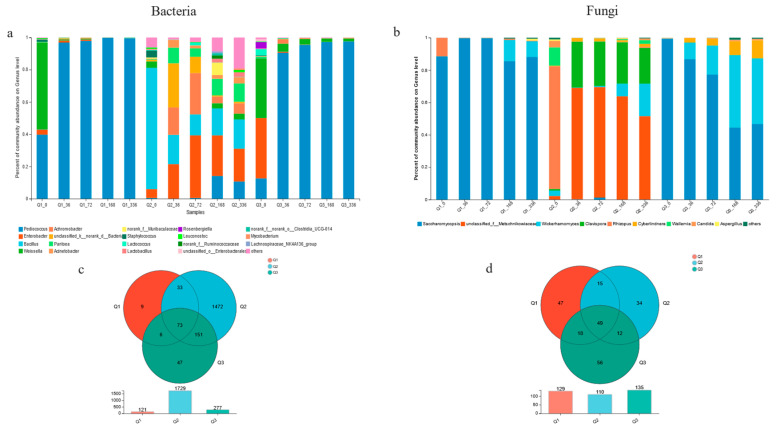
Relative abundance of microbial composition of genus (**a**,**b**) and Venn diagram of OUTs (**c**,**d**) in different Xiaoqus.

The Venn diagrams at the OUT level and abundance plots of microbial composition at the genus level are presented in Figure 2. It was evident that the bacterial OTUs in the Q2 samples were much greater than those in the other two samples, whereas the difference in fungal OTUs among all samples was minor, ranging from 110 to 135, which was in line with Yan’s research results [13]. The microbial composition and abundance of all Xiaoqu samples were distinct at the genus level. The microbial species composition of Q1 and Q3 starter (0 d) was similar, with *Saccharomycopsis* and *Rhizopus* being the predominant fungal species, and *Pediococcus*, *Weissella*, and *Enterobacter* being the primary bacterial species. During the fermentation process, the diversity of bacteria decreased, with *Pediococcus* being absolutely dominant, while the diversity of fungi increased (Q1: *Saccharomycopsis* and *Wickerhamomyces*); Q3 also showed the same trend. It is evident that *Pediococcus* was more suitable for growth and reproduction in black rice, as it utilized the nutrients in black rice to produce a large number of organic acids and bacteriocins, thereby inhibiting the growth of other bacteria and achieving “self-purification” [22,23]. Compared to the other two starters, the microbial species composition of Jiangxi Yingtan Xiaoqu was notably dissimilar, mainly composed of *Rhizopus*, *Wallemia*, *Clacispora*, *Bacillus*, and *Enterobacter*. Interestingly, the fermentation process was mainly characterized by three yeasts: unclassified_f_Metschnikowiaceae, *Clacispora*, and *Wickerhamomyces*, which mainly proliferated during the alcohol fermentation stage. Meanwhile, *Enterobacter*, *Bacillus*, *Achromobacter*, and *Pediococcus* were the bacteria most commonly found during this process. First observed in Chinese rice wine, unclassified_f_Metschnikowiaceae is a type of non-Saccharomyces yeast that can effectively enhance the flavor of red wine [24]. Additionally, it had a notable advantage over other yeasts when it came to the brewing of BRW. 

Dominant microorganisms with high abundance are believed to be major parts of food fermentation which are essential to the food fermentation process [25]; they are usually identified by having an average abundance of over 1% and existing in 50% of samples [16]. Table S3 shows that the dominant microbial communities of different product groups were not identical, yet there were similarities—*Pediococcus*, *Saccharomycopsis*, and *Wickerhamomyces* were present in the dominant microorganisms of both Q1 and Q3, while two dominant microbes of Q3 were *Weissella* and *Cyberlindera*. Additionally, two dominant microorganisms, *Pediococcus* and *Wickerhamomyces*, were present among the three Xiaoqu samples.

#### 3.3.3. Beta Diversity Analysis

The Principal Coordinate Analysis (PCoA) of microorganisms in all samples indicated that the Xiaoqu samples were distinctly dispersed in the coordinates (Figure S2), which highlighted the considerable regional variations in the microflora of these Xiaoqus (*p* < 0.01). Nevertheless, they still shared some common features, particularly Q1 and Q3, which had highly comparable microbial community structures. In terms of the fermentation process of BRW, each wine sample could be categorized into three stages: the initial starter stage (Xiaoqu), the saccharification stage (0–72 h), and the alcohol fermentation stage (72–336 h).

In order to gain a more comprehensive insight into the microbial community succession during the fermentation of BRW, the LEfSe was employed to analyze the biomarkers of three groups across three different fermentation stages (linear discriminant analysis, LDA > 4.0, *p* < 0.05). Every fermentation stage of different starters was characterized by biomarkers, and the biomarkers in the initial fermentation and saccharification stages were not alike (Table S4). The biomarkers found in Sichuan Dazhu Xiaoqu were classified as *Weissella* and *Rhizopus*, while its saccharification stage was attributed to the genus *Saccharomycopsis*; the Anhui Yingtan sample was marked by its biomarkers of *Bacillus* and *Rhizopus* in Xiaoqu, but *Enterobacter* and unclassified_f_Metschnikowiaceae during the saccharification stage. The characteristic genera *Weissella* and *Saccharomycopsis* were indicative of the Hubei Fangxian Xiaoqu, and *Achromobacter* was present in the saccharification stage. The discrepancy may be due to the dissimilar initial microbial composition of starters causing different biomarkers during the saccharification stage. Both Q1 and Q3 wine samples were identified as belonging to the genus *Saccharomycopsis* during the saccharification stage. It is important to recognize that *Pediococcus* and *Wickerhamomyces* were biomarkers in the alcohol fermentation stage of all samples.

### 3.4. Microbial Succession during Black Rice Wine Fermentation

**Figure 3:**
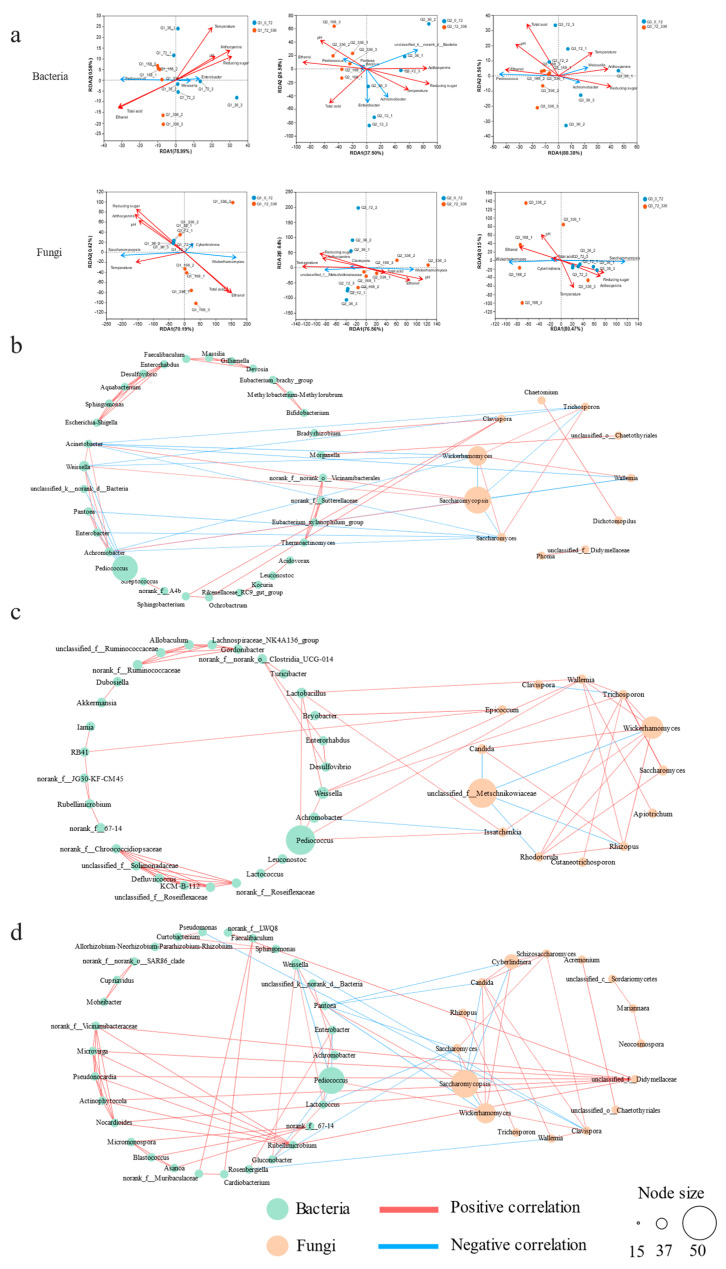
Microbial succession during black rice wine fermentation. (**a**) RDA analysis based on relative abundance of dominant genus, the red arrow signifies environmental factors, and the blue arrow symbolizes microorganisms; (**b**–**d**) represent microbial interaction network of Q1, Q2, and Q3, respectively, constructed using Spearman’s correlation (|r| > 0.8, *p* < 0.05).

The primary drivers of microbial community succession in the BRW brewing process comprise both non-biological and biological elements. These non-biological components involve temperature as well as metabolic byproducts such as reducing sugars, total acidity, ethanol, pH, and total anthocyanins, which can be regarded as critical environmental factors [16,26]. RDA was used to further analyze the relationship between key microorganisms and environmental factors (Figure 3a) [27]. The findings indicated a uniform link between microbial and environmental factors during all BRWs’ brewing processes, where *Pediococcus* and *Wickerhamomyces* had a negative correlation with temperature, reducing sugars, and total anthocyanins for all samples, but were positively associated with alcohol and total acidity. It had been observed that temperature can influence the fermentation process, particularly when the temperature drops to 20 °C in the later stages of alcohol fermentation. This could lead to a decrease in the physiological activity of *Pediococcus* [28], thus avoiding the total acids of the fermentation broth from increasing significantly, and consequently preventing the occurrence of wine rancidity. The *Wickerhamomyces* in the Q1 and Q3 samples and the unclassified_f_Metschnikowiaceae in Q2 displayed an inverse relationship with total acid and alcohol content, while exhibiting a positive correlation with reducing sugars. *Enterobacter* demonstrated a distinct response to environmental factors in the samples from Sichuan Dazhu Xiaoqu and Jiangxi Yingtan Xiaoqu: a negative correlation with alcohol and total acid was observed in Sichuan Dazhu, whereas only a negative correlation with alcohol and a positive correlation with total acid was seen in Jiangxi Yingtan.

The reason for this could be that microbial succession was affected not only by environmental conditions but also by the interactions between microorganisms (biological factors). By utilizing Spearman’s analysis, microbial network correlations between the species located in different Xiaoqus were evaluated (Figure 3b–d). In the Q1, Q2, and Q3 samples, 44, 42, and 46 nodes, respectively, were identified, along with 69, 67, and 78 pairs of correlations (|r| > 0.8, *p* < 0.05). The correlation between the bacterial and fungal communities was represented by 18, 9, and 18 pairs, respectively, while the bacterial and fungal communities each had 41, 38, and 41 pairs and 10, 20, and 19 pairs of correlations, respectively. The findings suggested a more intricate correlation between bacterial communities than fungi communities in different regions of Xiaoqu, and were in agreement with Xiao’s research results [16]. Furthermore, the interactions between bacterial and fungal communities were less significant than those within their respective communities, likely due to habitat filtration that restricted the magnitude of such interactions [29]. A high abundance of fungal strains (Q1: *Saccharomycopsis*, Q2: unclassified_f_Metschnikowiaceae, and Q3: *Saccharomycopsis*) found in the samples exhibited a competitive dynamic with other fungal strains such as *Wickerhamomyces* and *Saccharomyces*. Coupled with environmental factor analysis, it is hypothesized that *Saccharomycopsis* and unclassified_f_Metschnikowiaceae secreted glycosylases, proteases, and other enzymes to break down starch and protein into smaller molecules that were then used by *Saccharomyces* and *Wickerhamomyces* for reproduction and ethanol production [30]. However, the accumulation of ethanol eventually inhibited the growth of the low-alcohol-tolerance genus *Saccharomycopsis* and unclassified_f_Metschnikowiaceae. It is worth recognizing that strong symbiotic relationships existed between low-abundance bacterial or fungal genera in all samples, demonstrating that these low-abundance microorganisms may be critical for maintaining the microbial community structure and functional stability during BRW brewing [23,31]. 

### 3.5. Correlations between Core Functional Microbial Community and Characteristic Flavor Compounds

**Figure 4:**
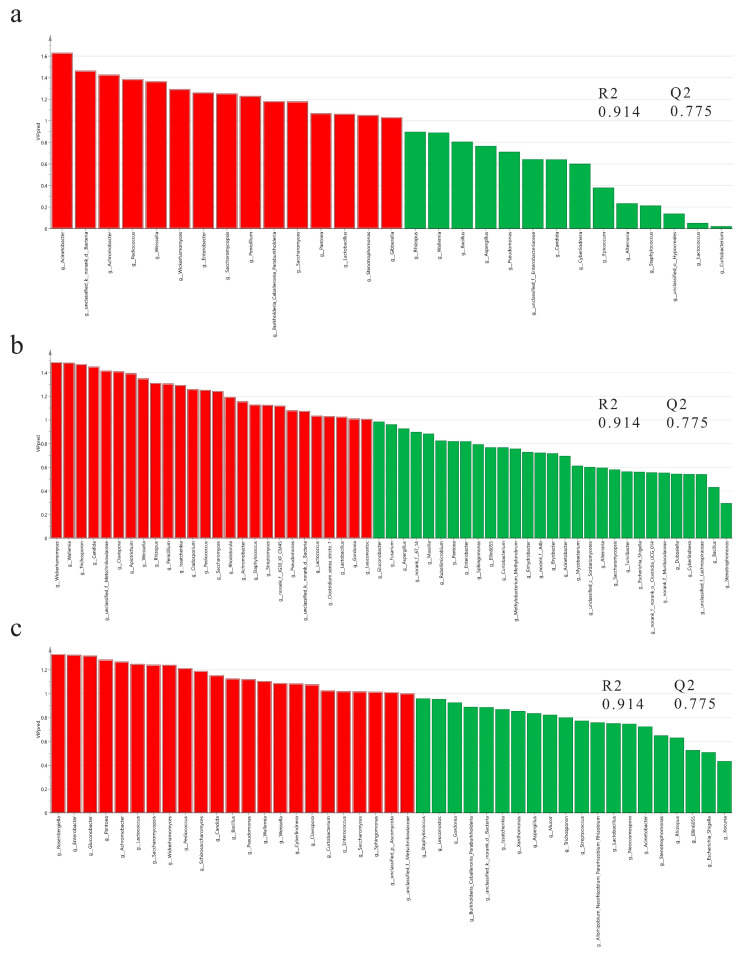

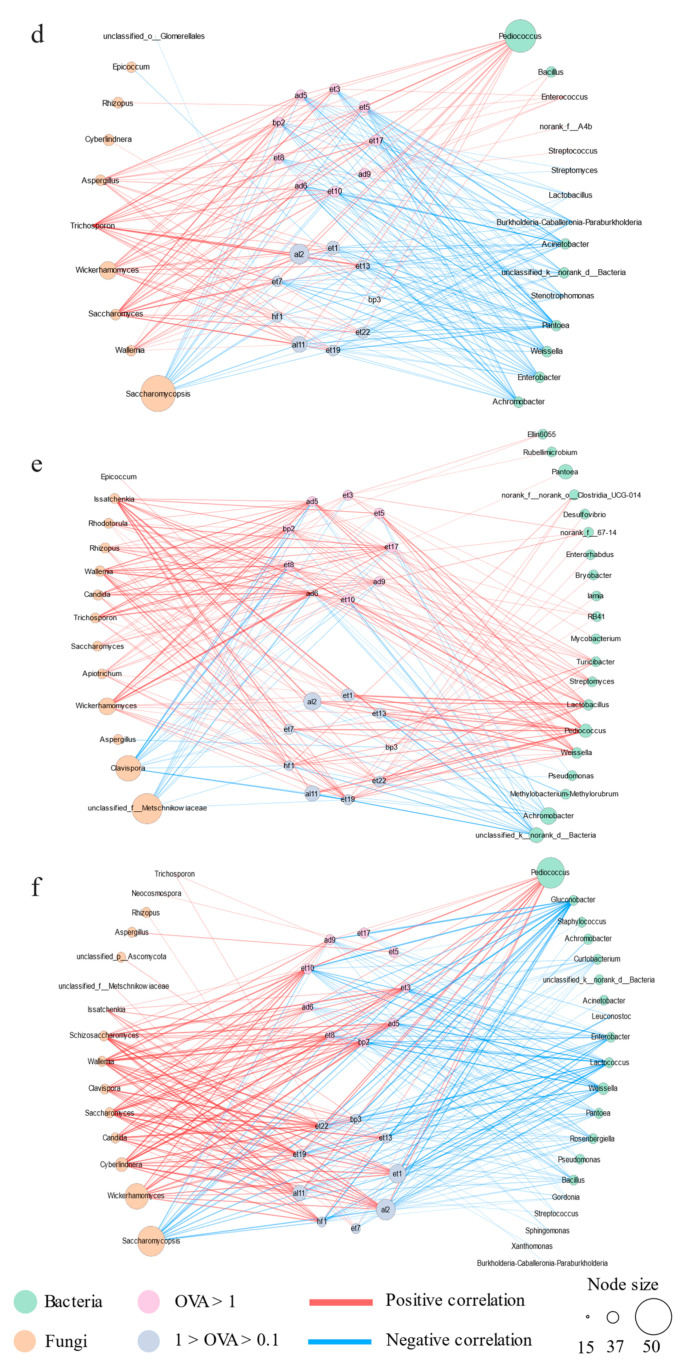
Correlation between microbial genera and volatile flavor compounds. VIP plot of the microbiota using O2PLS modeling during black rice wine fermentation of Q1 (**a**), Q2 (**b**) and Q3 (**c**) Xiaoqu (red, VIP > 1.0, *p* < 0.05); Correlation network of Q1 (**d**), Q2 (**e**) and Q3 (**f**) Xiaoqu were constructed using Spearman’s correlation coefficient (|r| > 0.6, *p* < 0.05) among the microbial community (20 top fungi and 50 top bacteria) and the 18 characteristic flavor substances (OAV ≥ 1: isoamyl acetate (et5), ethyl octanoate (et10), 2-octenal (ad5), ethyl decanoate (et17), 2-decenal (ad9), ethyl heptanoate (et8), ethyl butyrate (et3), and nonanal (ad6); 1 > OAV > 0.1: ethyl palmitate (et22), phenyl ethyl acetate (et13), phenyl ethanol (al11), limonene (hf1), ethyl acetate (et1), 4-ethyl guaiacol (bp3), isoamyl alcohol (al2), butyrolactone (et7), and ethyl cinnamate (et18)) in black rice wine.

It is widely accepted that the rice wine aroma has a significant influence on its sensory quality and consumer preferences [32]. The aromatic variations are mainly due to the raw materials, fermentation agents, and the fermentation and aging processes [15]. Nevertheless, in this research, the different flavor of the BRW was mainly caused by the diverse microbial compositions in starters. To explore the core functional microbial communities in the brewing process of BRW, the O2PLS model, based on variable importance in projection, was used to analyze the impact of microbial communities (20 top fungi and 50 top bacteria) on the formation of all volatile flavor compounds in all BRWs. The O2PLS method was appropriate for analysis and prediction, as demonstrated by the R^2^ and Q^2^ values, which were close to 1.0 and greater than 0.5, respectively [33]. There were 15, 26, and 23 genera, respectively, which had a significant influence on flavor substances in Q1, Q2 and Q3 (VIP > 1.0, *p* < 0.05) (Figure 4a–c and Table S5). While it is understood that certain strains played a major role in the development of volatile compounds in the BRW, it is yet to be determined which strains were beneficial for the formation of the wine’s characteristic flavor compounds, and which were negative. The Spearman’s correlation between the microbial community (20 top fungi and 50 top bacteria) and the 18 characteristic flavor substances of the BRW was calculated and then visualized using Cytoscape software, see Figure 4d–f. (|r| > 0.6, *p* < 0.05). It is observed that the dominant *Saccharomycopsis* in the samples from Sichuan Dazhu and Hubei Fangxian had a significant negative correlation with 16 and 15 characteristic flavor substances, respectively. Similarly, the high abundance fungi in the Jiangxi Yingtan sample, such as unclassified_f_Metschnikowiaceae and *Clavispora*, evinced a significant negative correlation with 16 characteristic flavor substances. On the basis of the prior analysis, it is speculated that the main function of these microbes is to decompose macromolecular substances and supply the necessary precursors for microorganisms that generate flavor. The presence of other fungi, including abnormal *Wickerhamomyces* and *Saccharomyces* genera, had been found to significantly contribute to the formation of characteristic flavor compounds in three BRWs. Viewed from the perspective of bacterial communities, in the Q1 and Q3 samples *Pediococcus* was significantly positively correlated with the characteristic flavor compounds, whereas *Achromobacter*, *Pantoea* and *Acinetobacter*, being low-abundance bacterial genera, displayed a negative correlation. A strong positive correlation was observed between lactic acid bacteria (*Lactobacillus*, *Weissella* and *Pediococcus*) and flavor compounds in Q2. An increased number of connections between one microbe and characteristic volatile substances in the network indicates that the microorganism is more vital for the formation of flavor compounds [34]. In addition, functional microorganisms are deemed to have a connectivity of more than 10 with characteristic volatile compounds [23]. Table S6 demonstrates that 11 fungal genera and 3 bacterial genera were selected as the functional microorganisms that produced characteristic volatile flavors, indicating that fungi were more influential in the fermentation process, which was consistent with previous research results [34,35].

To further clarify the core functional microbiota that plays a major role in characteristic flavor substances and the overall aroma flavor substances of BRW, screening would be based on the following criteria [36]: (1) the VIP value of the microbial genus was >1.0; (2) the correlation coefficient (r) between microbial genera and at least 10 characteristic flavor substances was >0.6 (*p* < 0.05). As a result (Table 2), the core functional microbiota responsible for producing aroma in Q1 were *Wickerhamomyces*, *Saccharomyces*, and *Pediococcus*; Q2 included *Wickerhamomyces*, *Issatchenkia*, *Wallemia*, *Pediococcus*, *Weissella*, and *Lactobacillus*; Q3 comprised *Schizosaccharomyces*, *Saccharomyces*, *Wickerhamomyces*, *Candida*, *Cyberlindnera*, *Wallemia*, *Clavispora*, and *Pediococcus*, with *Wickerhamomyces* and *Pediococcus* being the common functional strains. Generally, *Saccharomyces cerevisiae*, non-Saccharomyces yeasts and LAB were the major microorganisms that contributed to the flavor of BRW, and these were also found as core functional microbial groups in other fermented foods, such as Pu’er tea, soybean, etc. [23,37].

*Saccharomyces cerevisiae* can produce large amounts of ethanol, esters, and other flavor compounds [38]. Furthermore, it has been found to have a considerable positive correlation with the majority of volatile flavor compounds in this study. Yet, its contribution to ethanol and other volatile flavor compounds may be limited, due to its extremely low abundance (<0.1%). Consequently, when examining the core functional microorganisms responsible for producing characteristic volatile substances, it is essential to take into account both the abundance and functions [39]. Recent studies have revealed that non-Saccharomyces yeasts (such as *Wickerhamomyces*) may yield higher concentrations of alcohols and esters than *Saccharomyces cerevisiae*, and this study also discovered comparable results [40]. These implied that *Wickerhamomyces* was likely to be a major contributor to ethanol accumulation and the formation of volatile flavor compounds. 

Lactic acid bacteria, which include *Pediococcus*, *Weissella*, and *Lactobacillus*, are actively involved in many foods’ fermentation, not only imparting a pleasant flavor and nutritional value [41,42], but also restraining the growth of pathogenic bacteria and spoilage bacteria by producing antibacterial substances like lactic acid and bacteriocins [43,44], thereby achieving “self-purification” in the fermentation environment [23]. In this research, all Xiaoqus had varying amounts of food-borne pathogenic bacteria—*Enterobacter*—[45] but the extensive reproduction of LAB generated a large number of organic acids, thus reducing the number of *Enterobacter* and eventually eradicating them from the fermentation environment. 

To sum up, the production of black rice wine may involve two primary steps: (1) during the saccharification stage, *Saccharomycopsis* and unclassified_f_Metschnikowiaceae were responsible for breaking down macromolecular substances from various enzymatic hydrolysis materials, supplying precursor substances for the growth of bacteria and yeast. Simultaneously, LAB produced a great number of organic acids, resulting in the wine’s acidification, which not only impeded the growth of undesirable genera, but also created a suitable environment for the growth of yeast. (2) During the alcohol fermentation stage, both *Saccharomyces cerevisiae* and non-Saccharomyces yeasts were responsible for the production of ethanol and many volatile flavor components, ultimately resulting in BRW’s unique flavor. 

## 4. Conclusions

In this study, results indicated that the variation in the predominant microbial communities is attributed to regional disparities, resulting in considerable differences in physicochemical properties, volatile compounds and microbial succession during the BRW’s fermentation process, and thus producing different flavor profiles. A further examination showed the presence of 18 common characteristic flavor substances, and O2PLS and the Pearson correlation analysis verified the fact that *Saccharomyces cerevisiae*, non-Saccharomyces yeasts and LAB were the core functional microorganisms responsible for the flavor production of BRW. Consequently, future efforts should be directed toward exploring yeast and lactic acid bacteria, developing selected starters to enhance the controllability and quality of black rice wine brewing, and creating various flavors of black rice wines that meet the needs of target consumer groups.

## Figures and Tables

**Table 1:** OAVs of volatile compounds detected in endpoint samples of black rice wine.

	Compounds	Q1-336	Q2-336	Q3-336	Odor Description	T ^b^ (μg/L)
OVA > 1	Isoamyl acetate	201.77	125.65	66.16	Banana	3
	Octanoic acid ethyl ester	71.45	61.48	52.97	Fruity	5
	2-Octenal (E)-	25.80	17.72	14.66	Green	3
	Decanoic acid ethyl ester	16.76	15.31	13.58	Fruity	20
	2-Decenal	6.46	8.65	14.10	Green	2
	Heptanoic acid ethyl ester	5.27	2.90	5.27	Flowery	2
	Butanoic acid ethyl ester	5.24	5.41	2.47	Fruity	20
	Nonanal	3.87	3.66	6.70	Pungent	1
	Guaicol	1.39	3.87	3.44	Cooked black rice	10
	Pentanoic acid ethyl ester	1.70	-	-	Peach	3
	2-Heptenal	1.63	-	3.64	Green	5
	Butyric acid 2-methyl- ethyl ester	1.49	1.00	-	Fruity	18
	Benzeneacetaldehyde	1.21	-	-	Honey	4
	Isobutyric acid	-	-	2.43	Sour	20
1 > OVA > 0.1	Hexadecanoic acid ethyl ester	0.51	0.44	0.60	Waxy	1500
	Acetic acid 2-phenylethyl ester	0.47	0.54	0.11	Flowery	250
	Phenylethyl Alcohol	0.42	0.33	0.39	Flowery	10,000
	Limonene	0.38	0.33	0.44	Lemon	15
	Ethyl Acetate	0.28	0.24	0.77	Fruity, sweet	7500
	Phenol 4-ethyl-2-methoxy-	0.20	0.12	0.35	Buckwheat	20
	Isopentyl alcohol	0.09	0.07	0.11	Fusel	65,000
	Butyrolactone	0.15	0.16	0.20	Alcohol	35
	Tetradecanoic acid ethyl ester	0.11	0.10	0.16	Ether	2000
	Isobutyric acid	0.40	- ^a^	-	Sour	20
	Benzeneacetic acid ethyl ester	0.18	-	-	Flowery	73
	2-Octen-1-ol	-	-	0.36	Mushroom	20
	Decanal	-	0.21	0.29	Orange	10
	Caryophyllene	-	0.25	0.13	Woody	64
	Pentanoic acid ethyl ester	-	0.86	-	Fruity	3
	2-Heptenal	-	0.86	-	Green	5
	Benzeneacetaldehyde	-	0.88	0.75	Honey	4
	Butyric acid 2-methyl-ethyl ester	-	-	0.74	Fruity	18

^a^ “-”: not detected; ^b^ Odor thresholds taken from Reference [21].

**Table 2:** The core functional microbiota based on the O2PLS and Spearman’s analysis during black rice wine fermentation.

O2PLS-VIP > 1 and Spearman–Node Degree > 10	Q1	Q2	Q3
Fungi	*Wickerhamomyces*	*Wickerhamomyces*	*Schizosaccharomyces*
	*Saccharomyces*	*Wallemia*	*Saccharomyces*
		*Issatchenkia*	*Wickerhamomyces*
			*Candida*
			*Cyberlindnera*
			*Wallemia*
			*Clavispora*
Bacteria	*Pediococcus*	*Pediococcus*	*Pediococcus*
		*Weissella*	
		*Lactobacillus*	

## Data Availability

The data presented in this study are available onrequest from the corresponding author. The data are not publicly available due to restrictions eg privacy or ethical.

## Supplementary Materials

The following supporting information can be downloaded at: https://www.mdpi.com/article/10.3390/foods12193576/s1, Figure S1: VIPpred score chart of volatile components of different black rice wines (336 h) based on OPLS-DA analysis (VIPpred > 1 and *p* < 0.05). (a) Q1(blue) vs. Q2 (red); (b) Q3(blue) vs. Q2 (red); (c) Q3(blue) vs. Q1 (red); Blue and red compounds are biomarkers of relevant black rice wine; Figure S2: PCoA score plots of bacteria and fungi in all samples and each Xiaoqu samples; Table S1: Changes of physicochemical properties during black rice wine fermentation; Table S2: alpha diversity of black rice wine samples during fermentation; Table S3: The dominant microbial communities of different Xiaoqu during black rice wine fermentation; Table S4: Microbial biomarkers during the different fermentation stages. Table S5: The microbial genus by O2PLS modeling and the VIP > 1 during different black rice wine fermentation. Table S6: The function microbes thought Spearman’s correlation coefficient and node degree during black rice wine fermentation.
