# Supplementary material for: Diversifying the Flavor of Black Rice Wines through Three Different Regional Xiaoqus in China and Unraveling Their Core Functional Microorganisms
Source: Foods. 2023 Sep 26;12(19):3576. doi: 10.3390/foods12193576 (PMC10572163; doi:10.3390/foods12193576)
Supplement: Supplementary file 1 [file foods-12-03576-s001.zip › foods-2619901-supplementary.pdf]

Supplementary Materials

a

b

c

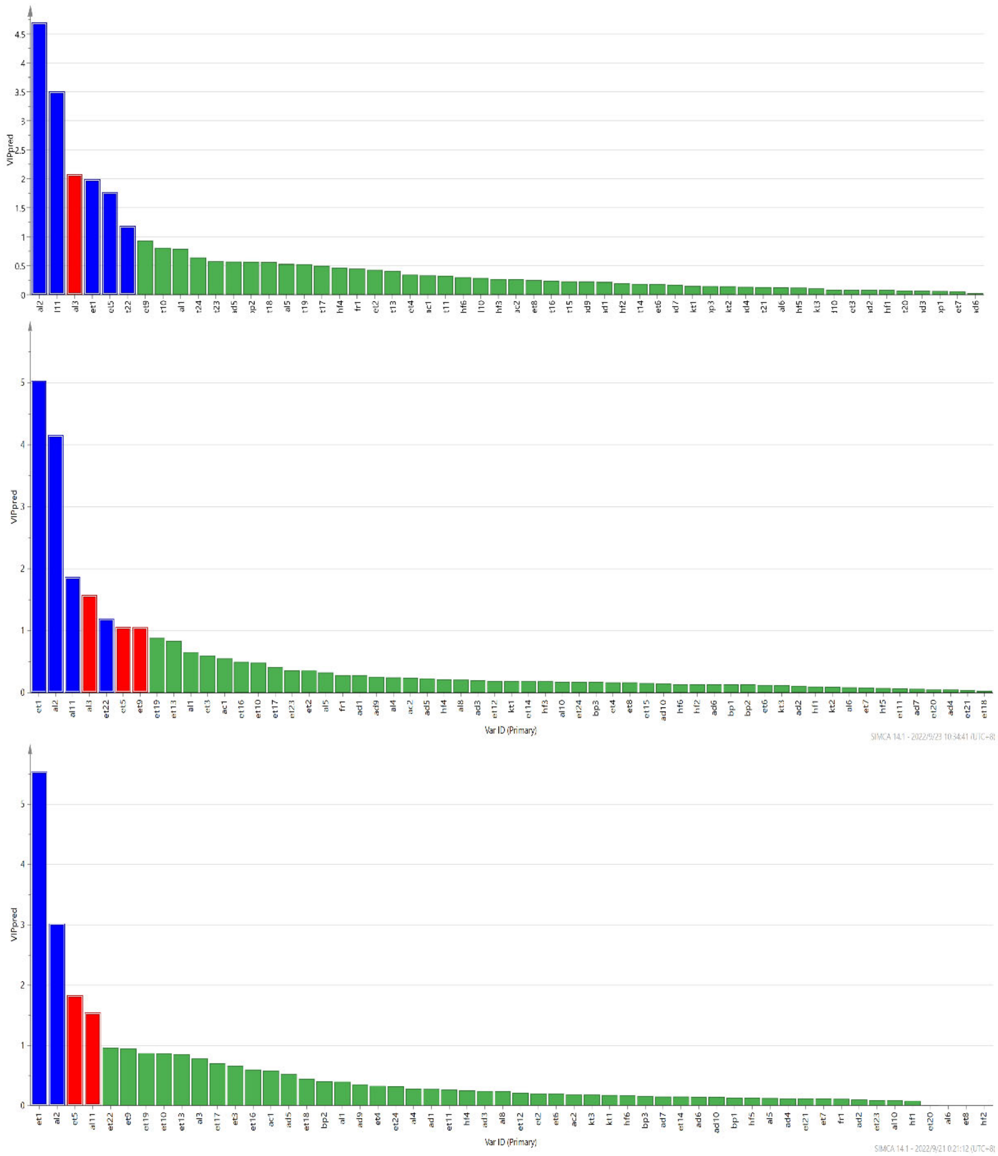

Figure S1. VIPpred score chart of volatile components of different black rice wines (336 h) based on OPLS-DA analysis (VIPpred > 1 and $p < 0.05$). (a) Q1(blue) *vs*. Q2 (red); (b) Q3(blue) *vs*. Q2 (red); (c) Q3(blue) *vs*. Q1 (red); Blue and red compounds are biomarkers of relevant black rice wine.

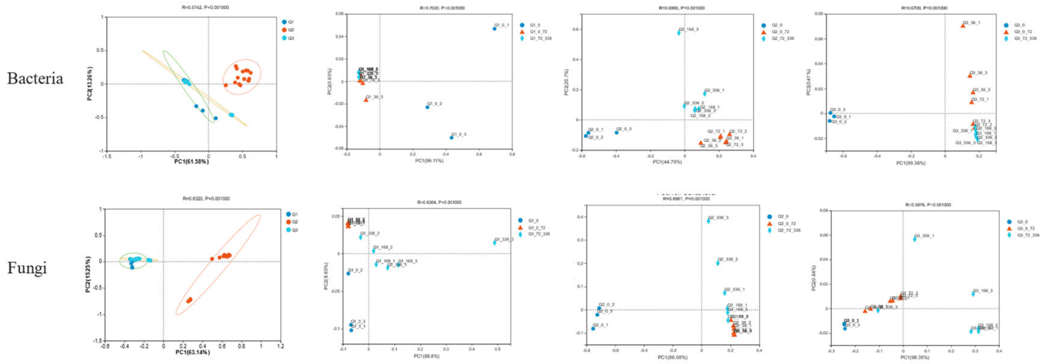

Figure S2. PCoA score plots of bacteria and fungi in all samples and each Xiaoqu samples.

Table S1. Changes of physicochemical properties during black rice wine fermentation .

Fermentation(h)	Total acids (g/L)			Reducing sugar(g/L)			Alcohol content (%, v/v)			Total anthocyanin content(mg/L)			pH		
	Q1	Q2	Q3	Q1	Q2	Q3	Q1	Q2	Q3	Q1	Q2	Q3	Q1	Q2	Q3
36	4.19± 0.03Ab	4.62± 0.09Bc	5.37± 0.1Ac	330.34± 14.77Cc	437.16± 15.2Ad	325.56± 13.11Bd	0.95± 0.05Aa	2.45± 0.05Ba	3.90± 0.00Ca	268.52± 2.52Aa	199.05± 2.34Ba	219.53± 3.66Ca	4.12± 0.02Ab	4.31± 0.04Bb	4.37± 0.02Ca
72	7.25± 0.04Ca	7.62± 0.11Bb	6.91± 0.06Ab	361.02± 11.05Cc	273.46± 15.81Ac	173.12± 6.41Bc	4.37± 0.06Ab	7.93± 0.06Bb	7.93± 0.12Bb	266.85± 0.33Bb	123.57± 2.34Cc	196.71± 2.03Ac	4.23± 0.03Ac	4.24± 0.01Aa	4.52± 0.02Bc
168	10.39± 0.09Ca	6.77± 0.04Aa	6.02± 0.06Ba	8.46± 0.70Cb	1.29± 0.06Ab	7.17± 0.4Bb	13.07± 0.21Ac	13.7± 0.26Bc	12.80± 0.10Ac	154.21± 3.46Cc	86.08± 1.47Bb	124.74± 4.14Ab	4.01± 0.01Aa	4.38± 0.04Bc	4.47± 0.02Cb
336	10.47± 0.04Ba	6.92± 0.04Aa	6.09± 0.02Ba	2.96± 0.01Ca	1.76± 0.01Aa	2.95± 0.11Ba	13.47± 0.06Ad	13.40± 0.10Ad	13.47± 0.06Ad	135.01± 2.18Cc	77.48± 1.01Bb	108.29± 3.06Ab	4.04± 0.04Aa	4.47± 0.02Bd	4.56± 0.04Cc

Q1 (Sicuang Dazhu), Q2 (Jiangxi Yingtan), Q3 (Hubei Fangxian); Values are presented as mean ± standard deviation (n = 3); A-B: Different capital letters mean the significant difference among the three Xiaoqu ($p < 0.05$); a-f: Different lowercase letters mean the significant difference among the same column ($p < 0.05$).

Table S2. alpha diversity of black rice wine samples during fermentation.

fermentation time (h)	Bacteria						Fungi						
	Q1		Q2		Q3		Q1		Q2		Q3		
	Shannon	Chao	Shannon	Chao	Shannon	Chao	Shannon	Chao	Shannon	Chao	Shannon	Chao	Goods
0	1.07	96.48	1.34	254.24	1.63	83.43	0.37	13	0.98	43.49	0.06	25.92	0.999
36	0.19	33.85	1.79	52.48	0.45	46.89	0.03	26.65	0.8	24.88	0.53	24.75	0.999
72	0.14	36.35	1.72	75.29	0.24	41.18	0.03	24.23	0.86	28.05	0.81	44.78	0.999
168	0.02	38.75	2.64	75.64	0.17	71.25	0.48	34.17	1.05	39.25	1.35	66.14	0.999
336	0.04	25.48	3.18	227.37	0.15	37.88	0.76	53.28	1.37	47.11	0.98	41.03	0.999

Table S3. The dominant microbial communities of different Xiaoqu during black rice wine fermentation.

Xiaoqu	Kingdom	Class	Genus	Abundance
Q1	Bacteria	Bacilli	*Pediococcus*	86.74%
	Fungi	Saccharomycetes	*Saccharomycopsis*	89.26%
	Fungi	Saccharomycetes	*Wickerhamomyces*	6.87%
Q2	Bacteria	Bacilli	*Bacillus*	28.18%
	Bacteria	Gammaproteobacteria	*Enterobacter*	22.30%
	Bacteria	Gammaproteobacteria	*Achromobacter*	10.62%
	Bacteria	unclassified_k__ norank_d__Bacteria	unclassified_k__ norank_d__Bacteria	8.07%
	Bacteria	Gammaproteobacteria	*Pantoea*	7.42%
	Bacteria	Bacilli	*Pediococcus*	5.14%
	Bacteria	Gammaproteobacteria	*Acinetobacter*	2.56%
	Bacteria	Bacilli	*Weissella*	2.20%
	Fungi	Saccharomycetes	unclassified _f__Metschnikowiaceae	49.68%
	Fungi	Saccharomycetes	*Clavispora*	20.38%
	Fungi	Saccharomycetes	*Wickerhamomyces*	7.93%

	Fungi	Saccharomycetes	*Cyberlindnera*	1.68%
Q3	Bacteria	Bacilli	*Pediococcus*	78.63%
	Bacteria	Bacilli	*Weissella*	9.92%
	Fungi	Saccharomycetes	*Saccharomycopsis*	74.99%
	Fungi	Saccharomycetes	*Wickerhamomyces*	19.53%
	Fungi	Saccharomycetes	*Cyberlindnera*	4.38%

Table S4. Microbial biomarkers during the different fermentation stages.

Xiaoqu	Fermentation stage	Kingdom	Order	Genus
Q1	Initial starter tage (0 h)	Bacteria	Lactobacillales	*Weissella*
		Fungi	Mucorales	*Rhizopus*
	Saccharification stage (0-72 h)	Bacteria	Xanthomonadales	–[a]
		Fungi	Saccharomycetales	*Saccharomycopsis*
	Alcohol fermentation stage (72-336 h)	Bacteria	Lactobacillales	*Pediococcus*
		Fungi	Saccharomycetales	*Wickerhamomyces*
Q2	Initial starter tage (0 h)	Bacteria	Bacillales	*Bacillus*
		Fungi	Mucorales	*Rhizopus*
	Saccharification stage (0-72 h)	Bacteria	Enterobacterales	*Enterobacter*
		Fungi	Saccharomycetales	unclassified _f__Metschnikowiaceae
	Alcohol fermentation stage (72-336 h)	Bacteria	Lactobacillales	*Pediococcus*
		Fungi	Saccharomycetales	*Wickerhamomyces*
Q3	Initial starter tage (0 h)	Bacteria	Lactobacillales	*Weissella*
		Fungi	Saccharomycetales	*Saccharomycopsis*
	Saccharification stage (0-72 h)	Bacteria	Burkholderiales	*Achromobacter*
		Fungi	-	-
	Alcohol fermentation stage (72-336 h)	Bacteria	Lactobacillales	*Pediococcus*
		Fungi	Saccharomycetales	*Wickerhamomyces*

[a] "–": not detected.

Table S5. The microbial genus by O2PLS modeling and the VIP > 1 during different black rice wine fermentation.

VIP > 1, $p < 0.05$	Q1	Q2	Q3
Fungi	*Wickerhamomyces*	*Wickerhamomyces*	*Saccharomycopsis*
	Saccharomycopsis	*Wallemia*	*Wickerhamomyces*
	Penicillium	*Trichosporon*	*Schizosaccharomyces*
	Saccharomyces	*Candida*	*Candida*
	Gibberella	unclassified_f__ Metschnikowiaceae	*Wallemia*
		Clavispora	*Cyberlindnera*
		Apiotrichum	*Clavispora*
		Rhizopus	*Saccharomyces*
		Penicillium	unclassified_p__ Ascomycota
		Issatchenkia	unclassified_f__ Metschnikowiaceae
		Cladosporium	
		Saccharomyces	
		Rhodotorula	
Bacteria	*Acinetobacter*	*Weissella*	*Rosenbergiella*
	unclassified_k__norank_d__ Bacteria	*Pediococcus*	*Enterobacter*
	Achromobacter	*Achromobacter*	*Gluconobacter*
	Pediococcus	*Staphylococcus*	*Pantoea*
	Weissella	*Streptomyces*	*Achromobacter*
	Enterobacter	norank_f__JG30_KF_CM45	*Lactococcus*
	Burkholderia_Caballeronia _Paraburkholderia	unclassified_k__norank_d__Bacteria	*Pediococcus*
	Pantoea	Clostridium_sensu_stricto_1	*Bacillus*

	Lactobacillus	*Lactococcus*	*Pseudomonas*
	Stenotrophomonas	*Pseudomonas*	*Weissella*
		Lactobacillus	*Curtobacterium*
		Gordonia	*Enterococcus*
		Leuconostoc	*Sphingomonas*

Table S6. The function microbes thought Spearman's correlation coefficient and node degree during black rice wine fermentation.

**r > 0.6 and Node Degree > 10**	**Q1**		**Q2**		**Q3**	
	**Genus**	**Node Degree**[a]	**Genus**	**Node Degree**	**Genus**	**Node Degree**
Fungi	*Wickerhamomyces*	16	*Wickerhamomyces*	17	*Schizosaccharomyces*	16
	Saccharomyces	16	*Wallemia*	16	*Saccharomyces*	15
	Trichosporon	16	*Issatchenkia*	15	*Wickerhamomyces*	15
	Aspergillus	14			*Candida*	15
	Issatchenkia	11			*Cyberlindnera*	15
					Wallemia	15
					Clavispora	13
Bacteria	*Pediococcus*	16	*Pediococcus*	15	*Pediococcus*	16
			Weissella	15		
			Lactobacillus	15		

[a]: Number of significant correlations between the microorganism and the characteristic aroma substances.
